# Evaluation of unplanned dialysis as a predictor of mortality in elderly dialysis patients: a retrospective data analysis

**DOI:** 10.1186/s12882-017-0778-0

**Published:** 2017-12-19

**Authors:** Debajyoti Roy, Anupama Roy Chowdhury, Shrikant Pande, Jia Wen Kam

**Affiliations:** 1 Changi General Hospital, 2 Simei St. 3, Singapore, 528889 Singapore; 2 Alexandra Health, 378 Alexandra Road, Singapore, 159964 Singapore

**Keywords:** Hemodialysis, Unplanned dialysis, Outcomes, Quality of life, Elderly

## Abstract

**Background:**

Increasing numbers of elderly patients are undergoing long-term dialysis. However, the role of dialysis in survival and quality of life is unclear, and poor outcomes may be associated with comorbidities rather than with age only. The initiation of unplanned dialysis in elderly patients with chronic kidney disease (CKD) has been reported to be associated with poor survival. We evaluated patient and practice factors associated with poor survival.

**Methods:**

We performed a retrospective analysis of 90 consecutive elderly patients (≥75 years) with CKD initiated on long-term dialysis at our renal unit between October 2010 and February 2014. Six patients were excluded; data from 84 remaining patients (≥75 years) with end-stage renal disease undergoing planned or unplanned dialysis were analyzed. Patients were followed up until death or January 2015. Patient factors such as age at initiation of dialysis and comorbidities (i.e., diabetes mellitus, ischemic heart disease [IHD], peripheral vascular disease, cancer, chronic obstructive pulmonary disease, and cognitive dysfunction) were analyzed. Practice factors such as planned or unplanned initiation of dialysis were compared in relation to survival outcomes. “Unplanned dialysis” was defined as a patient with known CKD stage 4 or 5 who had not been evaluated by a nephrologist in the 3 months before dialysis initiation.

**Results:**

The average age at dialysis initiation was 81.5 ± 4.5 years), serum albumin level was 24.8 ± 6 g/L, body mass index was 22.5 ± 4.8 kg/m^2^, and glycated hemoglobin A1c level was 6.3 ± 1.3. Overall, 51 (61%) and 33 (39%) patients underwent unplanned and planned dialysis, respectively. On univariate analysis, the presence of IHD, peripheral vascular disease, ≥3 comorbidities, and unplanned initiation of dialysis were significantly related to death. On multivariate analysis, unplanned start of dialysis, ischemic heart diseases and peripheral vascular disease remained significant. Survival rates at 3 and 12 months were 38.6% vs. 90.9% and 14.4% vs. 73.6% for unplanned vs. planned dialysis, respectively (*p* < 0.001). Unplanned dialysis was significantly associated with greater mortality.

**Conclusions:**

In elderly dialysis patients, unplanned start of dialysis was associated with poor survival. Patient characteristics such as associated peripheral vascular disease and IHD were associated with poor survival.

## Background

Previous studies have described discordance in survival data for elderly individuals. It is unclear whether age or the number of comorbidities has a greater impact on survival [1]. Little is also known about the impact of unplanned start of dialysis on survival [2–4]. This information is important, as with increasing life expectancy, more elderly individuals are undergoing dialysis. The role of dialysis in improving life expectancy and quality of life in this group of individuals is not clear. To receive dialysis, patients must visit hospitals or satellite centers, adding physical, emotional, and financial burdens on the patient and his or her family.

Diabetes mellitus (DM), peripheral vascular disease (PVD), ischemic heart disease (IHD), cerebrovascular accident (CVA), and chronic obstructive pulmonary disease (COPD) are the most common comorbidities associated with chronic kidney disease (CKD) and end-stage renal failure (ESRF). Many studies have suggested that multiple comorbidities rather than chronological age are associated with poor survival [5]. Differing survival data from various parts of the world may be due to selection bias in offering maintenance dialysis; hence, it is important to determine the relationship between comorbidities and survival.

A considerable proportion of elderly patients with CKD initiate dialysis in an unplanned manner. Often many of these patients need urgent dialysis initiation due to intercurrent acute medical or surgical illnesses. There is a paucity of literature addressing this issue. Thus, in the present study, we sought to evaluate patient and practice factors associated with poor survival.

## Methods

We performed a retrospective analysis of patients with CKD aged ≥75 years initiating long-term dialysis at Changi General Hospital in Singapore. We excluded patients hospitalized with acute kidney injury or patients with no prior health records. Of 90 consecutive patients who were initiated on long-term dialysis at our renal unit between October 2010 and February 2014, 6 patients were excluded, as they withdrew from dialysis and chose conservative medical therapy within 2 weeks of initiation. Data from 84 remaining patients were analyzed. Patients were followed up until death or January 2015. The SingHealth Institutional Review Board granted us approval to collect the retrospective data, and the requirement for informed consent was waived because of the study’s retrospective nature.

Data collection included demographic characteristics, such as the body mass index (BMI) and glycated hemoglobin (HbA1c) level, as well as comorbid conditions (coronary artery disease, PVD, cerebrovascular disease, diabetes, chronic lung disease, and cognitive dysfunction). Practice characteristics studied included the mode of dialysis initiation (planned versus unplanned) and type of dialysis access (arteriovenous fistula, arteriovenous graft, or central venous dialysis catheter). The primary outcome was death.

Definition of variables “Elderly” was defined as a patient aged 75 years and older. “Unplanned dialysis” was defined as a patient with known CKD stage 4 or 5 who had not been evaluated by a nephrologist within 3 months before initiation of dialysis.

### Statistical analysis

Univariate and Cox proportional hazards multivariate regression models were used to determine factors significantly associated with mortality. Univariate significant factors with *p* < 0.05 were then included and analyzed in the Cox Regression model. The Kaplan-Meier curve method was used to plot the survival curve. A two tailed, *p*-value of <0.05 was considered statistically significant. Stata Corp.2011, Stata Statistical software, release 12, Texas, USA was used.

## Results

We collected the following data for each patient: demographic details, mode of initiation of dialysis, survival, comorbidities, blood markers (i.e., albumin and HbA1c levels). We also collected information on the causes of death. Details of patients’ demographic characteristics and comorbidities are shown in Table 1.

**Table 1:** Characteristics of patients ≥75 years old at initiation of dialysis and comparison between acute unplanned and planned patients

	All	Acute Unplanned	Planned	*p*-value
Number of patients, n	84	51	33	
Mean Age (years) (±SD)	81.6 ± 4.5	81.7 ± 4.9	81.3 ± 3.7	0.709
Sex (Female)	63%	65%	61%	0.704
Body mass index (kg/m^2^)	22.5 ± 4.8	22.5 ± 4.5	22.4 ± 5.1	0.960
Serum albumin (g/L)	24.8 ± 6.0	24.2 ± 6.0	25.7 ± 5.9	0.267
HbA1c <6%	63%	73%	49%	0.026
Co-existing conditions (%)				
Diabetes mellitus (DM)	73%	75%	70%	0.629
Ischemic heart disease (IHD)	82%	90%	70%	0.017
Cerebrovascular accident (CVA)	35%	39%	27%	0.261
Peripheral vascular disease (PVD)	20%	31%	3%	0.002
Chronic obstructive pulmonary disease (COPD)	19%	28%	6%	0.015
Cancer	19%	20%	18%	0.871
Cognitive dysfunction	11%	12%	9%	> 0.999
Number of comorbidities^a^				< 0.001
None or one	19%	12%	30%	
Two	31%	19%	49%	
Three or more	50%	69%	21%	

^a^Comorbidities include DM, CAD, CVA, PVD, COPD and cancer

There were 84 patients enrolled in our study; 53 (63%) were women and 31 (37%) were men. Overall, 61% of patients were started on unplanned dialysis, and 39% were initiated on dialysis in a planned fashion.

At initiation of dialysis, the average age was 81.5 ± 4.5 years, serum albumin level was 24.8 ± 6 g/L, BMI was 22.5 ± 4.9 kg/m^2^, and HbA1c level was 6.30 ± 1.3%. Five patients (15%) were included in the planned dialysis group, and all of them had an arteriovenous fistula created; however, only one arteriovenous fistula was mature and could be used at the initiation of dialysis. The remaining 83 patients initiated dialysis with a central venous catheter.

Causes of death were classified as cardiac, infectious, malignant, and withdrawal of dialysis. Survival rates at 3 months and 12 months were 38.6% and 14.3%, respectively, for patients who began dialysis in an unplanned manner as compared to 90.9% and 73.6%, respectively, for those who began dialysis in a planned manner (log rank test, *p* < 0.001). The causes of death were IHD (37%), infectious pathology (42%), and withdrawal from dialysis (15%).

Results of univariate analysis showed that the presence of IHD (hazard ratio [HR] 3.05, *p* = 0.011), PVD (HR 2.55, *p* = 0.003) or having ≥3 comorbidities (HR 2.85, *p* = 0.013), and initiation of dialysis in an unplanned manner (HR 5.54, *p* = 0.001) were significantly related to death in patients undergoing hemodialysis (Table 2). However on the multivariate analysis IHD (HR 4.68, *p* = 0.005), PVD (HR 2.03, *p* = 0.045) and unplanned start of dialysis (HR 5.31, *p* < 0.001) were the only variables that remained significant (Table 3).

**Table 2:** Univariate predictors of mortality in patients ≥75 years old at initiation of dialysis

Variable	HR	95% CI	*p*-value
Mean Age (years) (±SD)	1.03	0.97–1.09	0.412
Sex (Male)	1.01	0.58–1.76	0.982
Diabetes mellitus	0.95	0.51–1.76	0.874
Ischemic heart disease	3.05	1.29–7.20	0.011
Cerebrovascular accident	1.25	0.72–2.18	0.424
Peripheral vascular disease	2.55	1.38–4.71	0.003
Chronic obstructive pulmonary disease	1.68	0.86–3.30	0.130
Cancer	1.03	0.53–2.01	0.923
Cognitive dysfunction	1.13	0.51–2.50	0.765
Number of comorbidities			
None or one	1.00	–	
Two	1.17	0.47–2.90	0.734
Three or more	2.85	1.25–6.47	0.013
Acute unplanned start of dialysis in the ICU	5.54	2.82–10.92	< 0.001

*HR* hazard ratio, *CI* confidence interval

**Table 3:** Multivariate predictors of mortality in patients ≥75 years old at initiation of dialysis

Variable	HR	95% CI	*p*-value
Ischemic heart disease	4.68	1.58–13.91	0.005
Peripheral vascular disease	2.03	1.02–4.04	0.045
Number of comorbidities			
None or one			
Two	0.50	0.17–1.45	0.204
Three or more	0.54	0.18–1.63	0.277
Acute unplanned start of dialysis in the ICU	5.31	2.44–11.56	< 0.001

*HR* hazard ratio, *CI* confidence interval

## Discussion

There is a paucity of data addressing the outcomes of elderly patients in whom dialysis is initiated in an unplanned manner, despite the fact that this group of patients is most likely to begin unplanned dialysis [2, 3].

With the increase in the aging population worldwide, the number of elderly people undergoing dialysis has been steadily increasing. Such people also have a considerable number of comorbidities and physical dependencies. Hemodialysis can lead to additional burdens, such as invasive procedures, extended time in the hospital, and high costs. The overall outcomes in the form of survival and quality of life are very relevant for this population [1].

Various definitions have been used to describe individuals who are “elderly” at the initiation of dialysis. For the purposes of this study, we defined “elderly” as those who were >75 years old at the initiation of dialysis.

Patients with ESRF who need dialysis should receive care in terms of a permanent vascular access well before the onset of dialysis. Despite this, unplanned dialysis often takes place in emergency settings [2]. Various terms are used in the literature to describe this occurrence, including unplanned dialysis, non-programmed, non-acute, or dialysis started under life-threatening conditions [2–4].

Survival rates and rates of complications can often be improved in patients who have been undergoing regular follow-up with a nephrologist and for whom careful counseling and education have been given prior to dialysis initiation. In contrast, unplanned hemodialysis performed in acute emergency situations is associated with increased complications and worse survival. Few studies have published survival outcomes in patients undergoing unplanned dialysis, and there are limited data available with respect to survival in very elderly patients undergoing unplanned dialysis. We therefore evaluated morbidity and mortality in this understudied patient group.

In our study, patients’ age was not associated with increased mortality (*p* = 0.73 0.95–1.07; Table 2).

Comorbidities, rather than age, have often been reported as having a greater effect on poor survival in elderly dialysis patients. The North Thames Dialysis Study addressed clinical outcomes, quality of life, and costs in a cohort of 221 elderly patients. The authors reported 1-year survival rates of 80%, 69%, and 54%, respectively, in patients aged 70–74 years, 75–79 years, and ≥80 years (*p* = 0.008). Except for poorer survival associated with PVD, they found no difference in outcomes in patients with comorbidities such as cancer, DM, late referral, IHD, CVD, or COPD. However, those with ≥2 comorbidities had poorer survival [6].

In an Australian study, based on ANZDATA, of 1781 elderly patients on dialysis, ≥3 associated comorbidities, late referrals (<3 months prior to dialysis), and an unprepared dialysis access were associated with poor survival [7]. Thus, survival of elderly dialysis patients appears to be predicted by both patient and practice characteristics [8].

A study in the United States reviewed survival data for patients undergoing dialysis, and the authors reported a 1-year mortality rate of 46% in both elderly and nonelderly populations. The patients in this study group had a relatively higher glomerular filtration rate and fewer comorbidities. Mortality was found to be associated with age, a non-ambulatory status, and comorbidities [9]. Schaefer et al. noted that 1-year and 5-year survival rates in elderly (>80 years of age) dialysis patients were 65% and 29%, respectively, and that patients spent nearly 10% of their lives in the hospital [5]. In our dataset, we noted poor survival in patients with IHD (HR 4.68, *p* = 0.005), PVD (HR 2.03, *p* = 0.045) and unplanned start of dialysis (HR 5.31, *p* < 0.001) (Table 3).

Unplanned dialysis has been variably defined in the literature as a patient who was referred late to renal services, a patient without a permanent access initiation of dialysis, or both [2, 3, 8, 10]. While the unplanned start of dialysis has been shown to be associated with increased mortality, it is common for elderly patients to start dialysis with a central venous catheter [11, 12]. In a cohort of patients undergoing dialysis in Ottawa, Canada, 41.3% were reported as having started dialysis in an unplanned fashion [13].

Metcalfe et al., in their study of 532 patients from the Scottish Renal Registry who started dialysis between October 1997 and September 1998, found that 129 (24%) started dialysis in an unplanned manner. They defined an unplanned start for patients with the last follow-up by a nephrologist occurring ≥1 month previously with no permanent dialysis access and who showed steady progression to ESRF. The unplanned start group had a 3.6-fold greater risk of death within 90 days of initiating dialysis than did those with a planned start to dialysis [10].

**Fig. 1:**
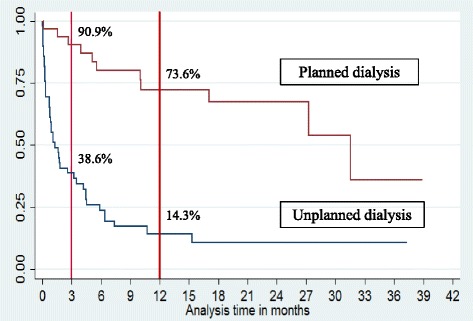
Kaplan-Meier suvival estimates: Planned dialysis vs unplanned dialysis

Our data is in agreement with the findings of Metcalfe, and we note poor survival in the unplanned dialysis group in comparison to the planned group with survival at 3 months of 38.6% vs. 90.9% and at 12 months of 14.3% vs. 73.6%. (Fig. 1).

In our planned dialysis group, five patients had an arteriovenous fistula created before initiation; however, only one arteriovenous fistula was mature and could be used. As a result, almost all our patients initiated dialysis with a central venous catheter. Due to socio-cultural factors, patients in our community have difficulty coming to terms with the need for dialysis and will often delay the decision to create a permanent vascular access.

Authors of retrospective studies of planned versus unplanned hemodialysis reported that patients with planned hemodialysis were younger, had a longer renal follow-up, and had more frequent medical visits [2, 3]. The planned hemodialysis group also had better patient education and biochemical and renal parameters at the beginning of dialysis [2, 3].

Similar findings with respect to better hemoglobin, calcium, and albumin parameters and lower urea, creatinine, and phosphate levels have been observed in other studies of patients undergoing planned hemodialysis [2, 14]. In contrast, unplanned hemodialysis initiation was associated with uremic symptoms, fluid overload, and more transfusion requirements. Such patients were also older and had a shorter follow-up (~3 months) [14, 15].

Compared to the available data, our mortality rates in this study were higher. The probable reason could be that for the majority of our study subjects, dialysis was initiated in an unplanned manner (e.g., for acute medical conditions or surgical emergency situations).

Limitations of study:

1. It is a single-center study.
2. Study design is observational.
3. However, the findings may reflect practice patterns in an East Asian population.

## Conclusions

In the present study, elderly patients with unplanned start of dialysis either in the intensive care unit or medical high dependency unit had poor survival. Poor survival in this group may be due to the intercurrent acute illness itself. Co-morbidities such as peripheral vascular disease or IHD were also associated with poor outcome.

## Abbreviations

BMI: body mass index
CKD: chronic kidney disease
COPD: chronic obstructive pulmonary disease
CVA: cerebrovascular accident
DM: Diabetes mellitus
ESRF: end-stage renal failure
HbA1c: glycated hemoglobin
HR: hazard ratio
IHD: ischemic heart disease
PVD: peripheral vascular disease
